# International Hahnemannian Association

**Published:** 1890-03

**Authors:** T. Dwight Stowe

**Affiliations:** Chairman; Bureau of Surgery; Mexico, New York


### INTERNATIONAL HAHNEMANNIAN ASSOCIATION.

### Bureau of Surgery, 1890.

The following gentlemen and ladies compose the Surgical Bureau of the I. H. A. for 1890:

Jas. B. Bell, M. D., 178 Commonwealth Ave., Boston, Mass.
Prof. E. Carleton, M. D., 53 West 45th St., New York.
Thos. M. Dillingham, M. D., 46 West 36th St., New York.
A. McNeil, M. D., 220 Turk St., San Francisco, Cal.
Miss E. J. Myers, M. D., 302 West 12th St., New York.

In addition to the above, very interesting articles are expected from Miss R. Dunlevy, House Physician of the Hospital for Women, 213 West 64th St., New York.

Up to this date the title of but one paper to be presented has been received, viz.: "Ulcers," by A. McNeil, of San Francisco, Cal. As it is frequently the case that the writing of articles for any bureau is necessarily postponed until a late date, I would very respectfully ask the several members of the Bureau of Surgery for 1890 to forward the titles of their respective theses to Samuel A. Kimball, M. D., 124 Commonwealth Ave., Boston, Mass., *on* or *before* June 1st, 1890. Also, if any member of this bureau be prevented from being present at the next meeting, to send his or her article to Dr. Kimball or myself, in time for presenting.

I wish to thank the gentlemen and Miss E. J. Myers, who compose the Bureau of Surgery, I. H. A., for 1890, for their courtesy and promptness in offering to constitute said bureau. Also, to Miss Dunlevy for her kind offer to furnish an article for our bureau. Knowing the ability of the several persons aforesaid, who constitute this bureau, I have no hesitation in stating that the Bureau of Surgery, I. H. A., 1890, will not be outdone by any of its predecessors.

Some of the members of this bureau think it very desirable to gather for a report at the next June meeting—which will convene at Newport, R. I.—reliable clinical experience in the treatment of venereal diseases, notably syphilis, including the use of the higher potencies. Therefore, a general invitation is herewith extended to all practitioners and students of pure Homœopathy to furnish anything that will "fill the bill" of our requirements for the June meeting.

Respectfully submitted,

T. Dwight Stowe, *Chairman.*

Bureau of Surgery.

Mexico, New York,

Feb. 21st, 1890.

